# Investigation of cell cytotoxic activity and molecular mechanism of 5β,19-epoxycucurbita-6,23(*E*)-diene-3β,19(*R*),25-triol isolated from *Momordica charantia* on hepatoma cells

**DOI:** 10.1080/13880209.2022.2077766

**Published:** 2022-06-27

**Authors:** Mei-Kang Yuan, Ju-Wen Kao, Wen-Tung Wu, Chiy-Rong Chen, Chi-I Chang, Yu-Jen Wu

**Affiliations:** a Department of Radiology, An Nan Hospital, China Medical University, Tainan, Taiwan; b Department of Medical Imaging and Radiology, Shu-Zen Junior College of Medicine and Management, Kaohsiung, Taiwan; c Department of Biological Science and Technology, Meiho University, Neipu, Taiwan; d Department of Food Science and Nutrition, Meiho University, Neipu, Taiwan; e Department of Life Science, National Taitung University, Taitung, Taiwan; f Graduate Institute of Biotechnology, National Pingtung University of Science and Technology, Neipu, Taiwan; g Yu Jun Biotechnology Co., Ltd., Kaohsiung, Taiwan

**Keywords:** Apoptosis, caspase, hepatocellular carcinoma cells, mitochondria, p38MAPK, JNK

## Abstract

**Context:**

*Momordica charantia* L. (Cucurbitaceae), known as bitter melon, is an edible fruit cultivated in the tropics. In this study, an active compound, 5β,19-epoxycucurbita-6,23(*E*)-diene-3β,19(*R*),25-triol (ECDT), isolated from *M. charantia* was investigated in regard to its cytotoxic effect on human hepatocellular carcinoma (HCC) cells.

**Objective:**

To examine the mechanisms of ECDT-induced apoptosis in HCC cells.

**Materials and methods:**

The inhibitive activity of ECDT on HA22T HCC cells was examined by MTT assay, colony formation assay, wound healing assay, TUNEL/DAPI staining, annexin V-fluorescein isothiocyanate/propidium iodide (PI) staining and JC-1 dye. HA22T cells were treated with ECDT (5, 10, 15, 20 and 25 μM) for 24 h, and the molecular mechanism of cells apoptosis was examined by Western blot. Cells treated with vehicle DMSO were used as the negative control.

**Results:**

ECDT inhibited the cell proliferation of HA22T cells in a dose-dependent manner. Flow cytometry showed that ECDT treatment at 10–20 μM increased early apoptosis by 10–14% and late apoptosis by 2–5%. Western blot revealed that ECDT treatment activated the mitochondrial-dependent apoptotic pathway, and ECDT-induced apoptosis was mediated by the caspase signalling pathway and activation of JNK and p38MAPK. Pre-treatment of cells with MAPK inhibitors (SB203580 or SP600125) reversed the ECDT-induced cell death, which further supported the involvement of the p38MAPK and JNK pathways.

**Discussion and conclusions:**

Our results indicated that ECDT can induce apoptosis through the p38MAPK and JNK pathways in HA22T cells. The findings suggested that ECDT has a valuable anticancer property with the potential to be developed as a new chemotherapeutic agent for the treatment of HCC.

## Introduction

Hepatocellular carcinoma (HCC), the most common type of primary liver cancer, accounts for between 85% and 90% of primary liver cancers. It is most commonly associated with cirrhosis and hepatitis (Leineweber et al. 2020). Primary liver cancer is the most prevalent cancer worldwide, and ranks the leading cause of cancer death due to its poor prognosis and survival rates (Bosch et al. 2004). HCC possesses several interesting epidemiologic features, including dynamic temporal trends, noticeable variations by geographic distribution, racial and ethnic groups, sex and age distribution, and the presence of several well-documented environmental, potentially preventable, risk factors (Fattovich et al. 2004). Cancer results when normal cells start to accumulate genetic errors and grow without control; death occurs when abnormal cells spread to other tissues. This group of diseases claims millions of lives each year, and remains one of the leading causes of death worldwide (El-Serag 2012).

Plants produce highly reactive and bioactive phytochemicals that have been demonstrated to be effective in protecting humans against diseases. The beneficial effects of intake of antioxidant-rich foods against diseases such as cancer, diabetes, cardiovascular diseases and other oxidative stress-related chronic diseases have been well-documented by a number of researchers (Sarker et al. 2020). A popular edible pod vegetable, *Momordica charantia* L. (Cucurbitaceae) (Johnson 2004), has multiple beneficial properties acting against human diseases: antiviral and antibacterial effects to alleviate pathogen infections; immunomodulatory effects to modify and regulate immune functions; a topical treatment for expelling intestinal gas; and the capacity to treat skin problems such as eczema, scabies and itchy rashes (Tan et al. 2008). Furthermore, the plant *M. charantia* contributes antidiabetic, abortifacient, anthelmintic and contraceptive effects (Das et al. 2006).

In the previous study, researchers discovered that a substance extracted from *M. charantia* fruit inhibits the growth of breast cancer cells through modulating signal transduction pathways (Ray et al. 2010). From *in vitro* studies and other findings from animal testing, accumulated data showed that several specific components isolated from *M. charantia* have valuable effects to promote human health (Jia et al. 2017; Bortolotti et al. 2019). A potential anti-herpes virus effect exerted by α- and β-momorcharin, and anticancer activity possessed by momordin enhance the pharmaceutical value of *M. charantia* (Fang et al. 2012). Some studies have shown that the apoptosis-inducing activity of leaf extract of *M. charantia* on several human cancer cells is initiated through caspase- and mitochondria-dependent pathways (Li et al. 2012). Matrix metalloproteinases (MMPs) are key for uncontrolled cancer cell proliferation and metastasis (Chowdhury et al. 2016), and extracts of *M. charantia* have been shown to prevent the unregulated secretion of MMPs (Jia et al. 2017). Bioactive compounds from *M. charantia* that have effects against numerous cancers have been isolated, researched and analysed, and their anticancer potential demonstrated (Kilcar et al. 2020). MAP30 isolated from *M. charantia* is a ribosome-inactivated protein and chemical analogue considered a potential therapeutic candidate against breast carcinomas (Song et al. 2018). When tested on human lung adenocarcinoma CL1-0 cells, a methanol extract of *M. charantia* induced cell apoptosis through mitochondria- and caspase-dependent pathways, while alterations of the Bcl-2 family were observed, including down-regulated antiproapoptotic Bcl-2 and up-regulated proapoptotic Bax (Li et al. 2012).

**Figure 1.**
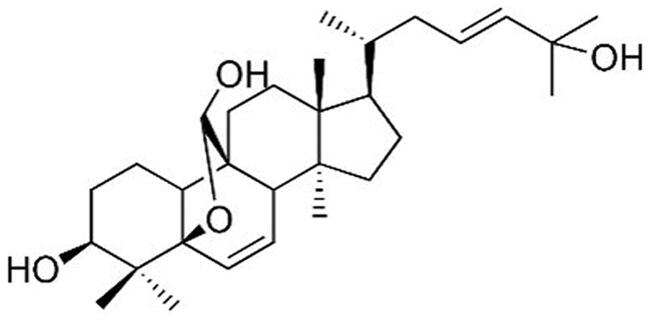
Structure of 5β,19-epoxycucurbita-6,23(*E*)-diene-3β,19(*R*),25-triol (ECDT) isolated from *Momordica charantia*.

The aim of the current study was to examine a novel compound isolated from bitter melon in order to explore and develop a novel effective treatment for HCC. This study focussed on the cytotoxic activity of 5β,19-epoxycucurbita-6,23(*E*)-diene-3β,19(*R*),25-triol (ECDT, shown in Figure 1), isolated from the fruit of *M. charantia*, against HCC HA22T cells. A cytotoxic effect of ECDT on HA22T cells was established from analytical data collected via experiments to examine the anti-proliferative and anti-migratory activities against HA22T cells after treatment with ECDT, and via observations of cell apoptosis by fluorescent imaging during ECDT treatment. In light of these results, valuable and practical information from the biochemical aspect derived from the cytotoxic effect of ECDT on HA22T cells could inform pre-clinical and clinical work to boost pharmaceutical development and continue to advance monitoring of human HCC.

## Materials and methods

### Reagents and antibodies

Rabbit anti-human antibodies were purchased from Cell Signaling Technology (Danvers, MA) to probe the expression of caspases (caspase-8, caspase-9, cleaved-caspase-9 and caspase-3, cleaved-caspase-3), Bcl-2 family members (Bcl-2, Bcl-xl, Mcl-1 and Bad) and the sub MAPK family (p38MAPK and *p*-p38MAPK) by Western blot. Rabbit anti-human antibodies manufactured by Epitomics (Burlingame, CA) were selected to probe the protein expression levels of cytochrome *C*, *p*-Bad and Bax by Western blot. ProteinTech Group (Chicago, IL)-manufactured rabbit anti-human JNK, *p*-JNK, ERK and *p*-ERK antibodies were employed to evaluate the expression changes of the corresponding antigens via Western blot. Cationic dye JC-1 was purchased from Biotium (Hayward, CA). 3-(4,5-Dimethylthiazol-2-yl)-2,5-diphenyltetrazolium bromide (MTT), Z-DEVD-FMK (caspase-3 inhibitor), Z-VAD-FMK (caspase-9 inhibitor), PD98059 (ERK-specific inhibitor), SB203580 (p38-specific inhibitor), SP600125 (JNK-specific inhibitor) and dimethyl sulphoxide (DMSO) antibodies were purchased from Sigma (St. Louis, MO). An annexin V-FITC apoptosis detection kit was purchased from Pharmingen (San Diego, CA).

### Source, purification and structural determination of 5β,19-epoxycucurbita-6,23(*E*)-diene-3β,19(*R*),25-triol

Fruits of *M. charantia* were purchased from contracted farmers in Kaohsiung, Taiwan. Identification of the voucher specimens was performed by Prof. Sheng-Zehn Yang, Curator of the Herbarium, National Pingtung University of Science and Technology. A voucher specimen was deposited in the laboratory of Dr. Chi-I Chang, Department of Biological Science and Technology, National Pingtung University of Science and Technology, Pingtung, Taiwan.

Dried fruit (30 kg) powder of *M. charantia* wild variant WB24 was extracted five times with 100% methanol (60 L) at room temperature (seven days per extraction). The combined methanol extract was filtered and then evaporated under reduced pressure to afford a black residue, which was suspended in H_2_O (4 L) and partitioned sequentially using ethyl acetate (EA) and *n*-butanol (*n*-BuOH) (5 × 4 L). The *n*-BuOH fraction (966 g) was chromatographed on a Diaion HP-20 column (150 × 10 cm) and eluted using mixtures of water and methanol of reducing polarity as eluents. Twenty-five fractions were collected as follows: fr. 1 (9000 mL, water), fr. 2 [6000 mL, water–methanol (98:2)], fr. 3 [6000 mL, water–methanol (95:5)], fr. 4 [6000 mL, water–methanol (93:7)], fr. 5 [6000 mL, water–methanol (90:10)], fr. 6 [6000 mL, water–methanol (88:12)], fr. 7 [6000 mL, water–methanol (85:15)], fr. 8 [6000 mL, water–methanol (83:17)], fr. 9 [6000 mL, water–methanol (80:20)], fr. 10 [7000 mL, water–methanol (75:25)], fr. 11 [7000 mL, water–methanol (70:30)], fr. 12 [7000 mL, water–methanol (65:35)], fr. 13 [7000 mL, water–methanol (60:40)], fr. 14 [7000 mL, water–methanol (55:45)], fr. 15 [7000 mL, water–methanol (50:50)], fr. 16 [1000 mL, water–methanol (45:55)], fr. 17 [7000 mL, water–methanol (40:60)], fr. 18 [7000 mL, water–methanol (35:65)], fr. 19 [7000 mL, water–methanol (30:70)], fr. 20 [7000 mL, water–methanol (25:75)], fr. 21 [7000 mL, water–methanol (20:80)], fr. 22 [7000 mL, water–methanol (15:85)], fr. 23 [7000 mL, water–methanol (10:90)], fr. 24 [7000 mL, water–methanol (5:95)] and fr. 25 (10,000 mL, methanol). Fraction 16 (6.1 g) was further chromatographed on a Sephadex LH-20 column (5 × 50 cm) with gradient elution (water–methanol, 1:1 to 0:1) to yield six fractions (800 mL each), frs 16A–16F. Fr. 16E (0.9 g) was subjected to column chromatography over Si gel with elution by CH_2_Cl_2_–MeOH (1:0 to 3:1) and semipreparative HPLC with elution by hexane–EA (6:4) to obtain compound 5β,19-epoxycucurbita-6,23(*E*)-diene-3β,19(*R*),25-triol (16 mg; ECDT), the chemical structure of which was identified by comparing the physical and spectral data (specific rotation, MS and NMR) with the values described in the literature (Liao et al. 2012).

5*β*,19-Epoxycucurbita-6,23(*E*)-diene-3*β*,19(R),25-triol: amorphous white powder; ^1^H NMR (400 MHz, CDC1_3_) *δ*: 0.83 (3H, s, H-30), 0.85 (3H, s, H-18), 0.86 (1H, d, *J*= 6.4 Hz, H-21), 0.87 (3H, s, H-29), 1.20 (3H, s, H-28), 1.29 (3H x 2, s, H-26, H-27), 2.54 (1H, br. t, *J*= 8.8 Hz, H-10), 2.82 (m, H-8), 3.40 (1H, m, H-3α), 3.70 (1H, d, *J*= 9.6 Hz, 3-OH), 5.10 (1H, d, *J*= 7.8 Hz, H-19), 5.58 (2H, m, H-23), 5.58 (2H, m, H-24), 5.65 (1H, dd, *J*= 4.0, 9.6 Hz, H-7), 6.06 (1H, dd, *J*= 2.0, 9.6 Hz, H-6); ^13^C NMR (100 MHz, CDCl_3_) *δ*: 14.7 (C-18), 17.2 (C-1), 18.6 (C-21), 19.7 (C-30), 20.4 (C-28), 23.1 (C-2), 23.9 (C-29), 27.1 (C-27), 27.9 (C-16), 29.8 (C-26), 30.0 (C-22), 30.5 (C-11), 33.5 (C-15), 36.1 (C-20), 37.1 (C-13), 39.0 (C-12), 41.3 (C-8), 45.0 (C-4), 48.0 (C-14), 48.4 (C-9), 50.0 (C-17), 70.7 (C-25), 76.1 (C-3), 85.0 (C-5), 105.3 (C-19), 125.2 (C-23), 132.4 (C-7), 132.6 (C-6), and 139.5 (C-24). EI-MS (70 eV) *m/z* (rel. int.): 426 [M–HCOOH]^+^ (31), 408 (22), 375 (19), 339 (25), 309 (24), 293 (24), 281 (17), 229 (23), 192 (39), 187 (43), 172 (100), 157 (52), 109 (77), 81 (49), 69 (46), and 55 (53).

### Cell culture and MTT assay

HA22T cells, a HCC cell line, were cultured in Dulbecco’s modified Eagle’s medium (DMEM) supplemented with 10% foetal bovine serum (FBS), 100 µg/mL streptomycin and 100 units/mL penicillin in a humidified 5% CO_2_ incubator at 37 °C. The proliferation of HCC cells was examined in order to determine the cytotoxicity of ECDT. DMSO was used as the control and for dissolving ECDT. ECDT at 5, 10, 15, 20 and 25 μM was prepared and applied to HA22T cells, then stored for 24 h for further use. A lack of mitochondrial reductase in dead cells indicates loss of the ability to transfer yellow MTT to blue formazan crystals; therefore, the concentration of blue formazan is an indicator of cell viability. HA22T cells (1 × 10^5^/well) were seeded in 96-well plates and treated with various concentrations of ECDT (5, 10, 15, 20 and 25 µM). After 24 h of incubation, MTT solution (0.5 mg/mL in PBS) was added to each well. The plates were incubated for 4 h at 37 °C, then the culture medium was removed, following which the cells were dissolved in 0.2 mL DMSO. The absorbance was measured at 595 nm using a microplate ELISA reader (Bio-Rad, Hercules, CA), and DMSO was used as the control. Samples from three repeated experiments were analysed for all experiments.

### Wound healing assay

Cells were seeded and allowed to grow and form a monolayer. A scrape in a straight line was then made across the culture using a tip. Cells were treated with various concentrations (0, 5, 10, 15 and 20 μM) of ECDT and allowed to migrate for 24 h. The migrated cells were observed and counted at time intervals of 6, 12 and 24 h under a microscope at ×100 magnification.

### Apoptotic progression analysed by flow cytometry

Cells were stained with annexin V-FITC coupled with propidium iodide (PI) to identify cell apoptotic stages by flow cytometry. 1 × 10^6^ cells were seeded onto 5-cm Petri dishes, then treated with various concentrations (0, 5, 10, 15 and 20 μM) of ECDT for 24 h, followed by the addition of 10 μg/mL annexin V-FITC and 20 μg/mL PI at 37 °C for 30 min. Apoptotic progression was analysed by flow cytometry and the results analysed using Cell-Quest software designed by Becton-Dickinson (Mansfield, MA).

### Immunofluorescence microscopy

HA22T cells (1 × 10^6^/well) were pre-treated with various concentrations (0, 5, 10, 15 and 20 μM) of ECDT for 24 h and maintained at a constant temperature (37 °C) in an incubator under 5% CO_2_. The DeadEnd™ Fluorometric TUNEL System (Promega, Madison, WI) was used to detect DNA fragmentation. Cleavage of DNA during apoptosis exposes the free 3′-OH DNA end, which is labelled with fluorescein-12-dUTP in an enzymatic reaction. Fluorescence microscopy (Chinetek Scientific, Hong Kong) was performed to visualize the fluorescein-labelled DNA to elucidate the progression of DNA damage. DAPI was incorporated to confirm the apoptotic progression resulting from TUNEL. With the assistance of fluorescence microscopy, images of the condense nuclei were obtained and analysed.

### Fluorescence microscopy

Changes in the mitochondrial membrane potential (Δ*Ψ*m), as an indication of deterioration of cells after ECDT treatment, were examined according to the reaction with cationic dye JC-1. JC-1 accumulates in healthy mitochondria and appears as red fluorescence (560 nm). When the mitochondrial membrane potential collapses, JC-1 uptake is limited to the cytoplasm, where it yields green fluorescence (530 nm). By means of fluorescence imaging, apoptotic cells can be easily and clearly distinguished from non-apoptotic cells. HA22T cells (1 × 10^6^/well) were pre-treated with various concentrations (0, 5, 10 and 20 μM) of ECDT for 24 h in a constant 37 °C incubator under 5% CO_2_. Then, following the addition of 10 mg/mL JC-1 dye, and two subsequent washes with PBS, red/green fluorescence images were observed by fluorescence microscopy, indicating the health condition of the cells of interest.

### Western blot analysis

Twenty micrograms of each lysate obtained from different concentrations (0, 5, 10 and 20 μM) of ECDT were separated by 12.5% SDS-polyacrylamide gel electrophoresis, then transferred or blotted onto PVDF membrane (Millipore, Burlington, MA) at 400 mA by Transphor TE 62 (Hoefer, Holliston, MA). A blocking reaction was initiated by incubation with 5% milk in PBST. Next, the membranes were stained with antibodies specific to the target proteins at 4 °C for 2 h or overnight. PBST was then used to wash the membranes for five minutes, repeated three times, to minimize background and remove unbound antibodies. Horseradish peroxidase (HRP)-conjugated antibodies (1:5000) were added for 1 h to detect the tested material on the membranes. The location and intensity of the specific reaction were revealed after incubation with the colourless substrate, which could then be visualized.

### Statistical analysis

All values were presented as means ± standard deviation (S.D.) from three independent experiments performed in triplicate. Student’s *t*-test was employed (Sigma-Stat 2.0, San Rafael, CA). A *p* value <0.05 was considered statistically significant.

## Results

### Repression of cell proliferation and migration of HA22T cells by ECDT

**Figure 2.**
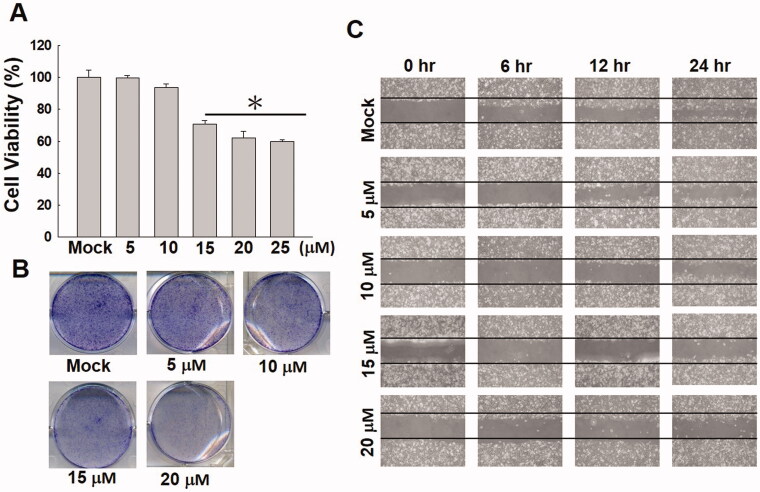
Activity of ECDT in repressing cell proliferation and migration in HA22T cells. (A) Cell viability was dose-dependently suppressed after ECDT treatment (15–25 μM) for 24 h (*p< 0.01) in an MTT assay. (B) Changes in morphology of HA22T cells treated with ECDT in a colony formation assay observed under inverted light microscopy. (C) HA22T cells treated with increasing concentrations of ECDT exhibited acceleration in the degree of suppression of cell migration ability. Mock: cells treated with vehicle control (DMSO).

A potential cytotoxic effect of 5β,19-epoxycucurbita-6,23(*E*)-diene-3β,19(*R*),25-triol (ECDT) on HA22T cells was determined by MTT, colony formation assay and cell migration assay. HA22T cells were treated with various concentrations (5, 10, 15, 20 and 25 μM) of ECDT for 24 h. The results shown in Figure 2(A) confirm that the HA22T cell viability was significantly repressed by treatment with ECDT in a dose-dependent manner. The cytotoxic efficacy of ECDT was enhanced by increasing the concentration of ECDT (15–25 μM). The morphology of the HA22T cells was examined by colony formation assay and the results observed under an inverted light microscope, which showed a decrease in the cell population following ECDT treatment. Colonies of HA22T cells formed presented sparingly at concentrations of 15 and 20 μM, in comparison with the more abundant thriving colonies formed by cells without ECDT treatment (Figure 2(B)). The observations made from the wound healing assay demonstrated the ability of ECDT to suppress cell migration in dose-dependent and time-dependent manners (Figure 2(C)).

### ECDT treatment initiated the apoptotic process in HA22T cells

**Figure 3.**
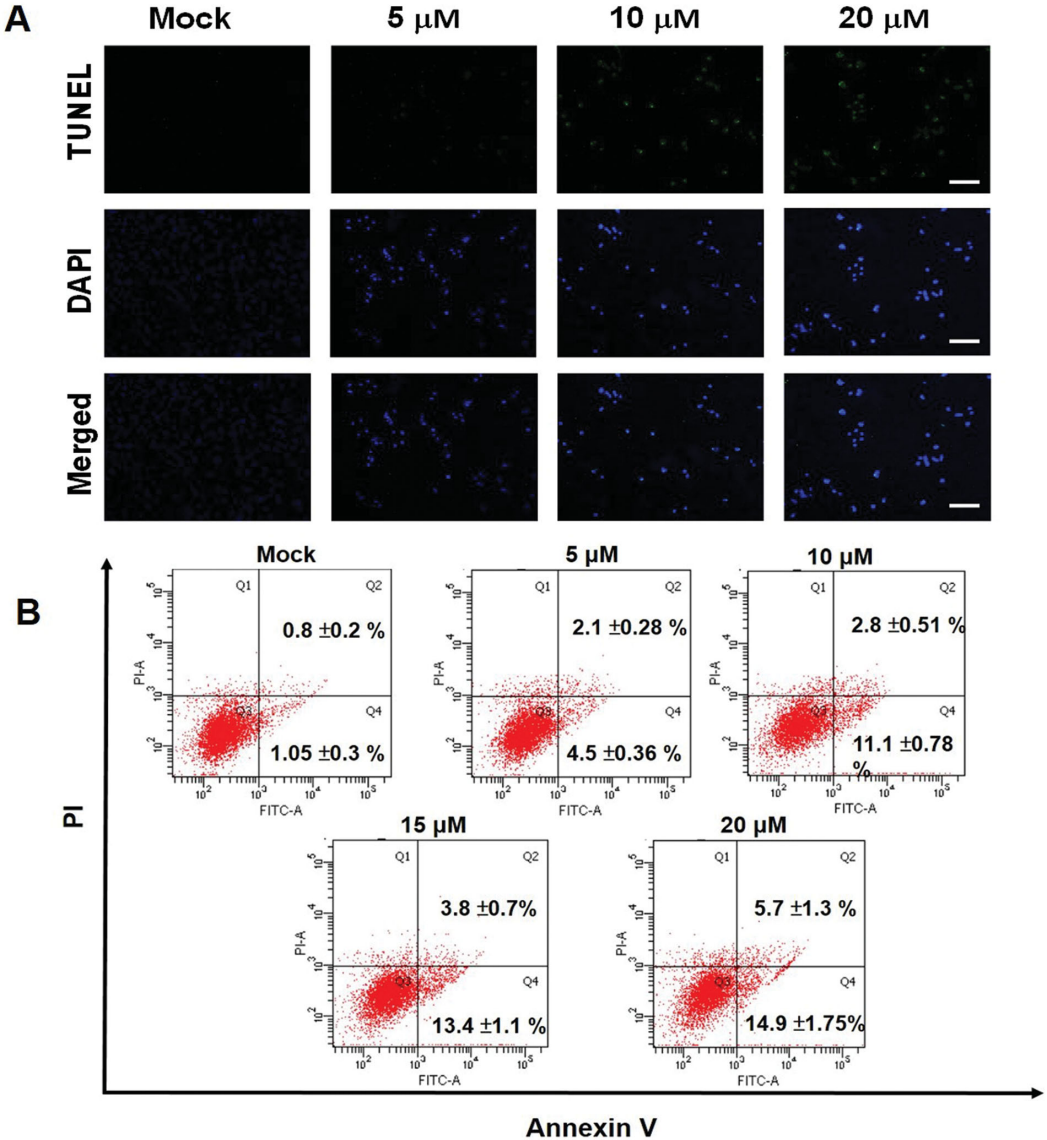
Apoptotic stages of ECDT-treated HA22T cells. (A) Fluorescence imaging of apoptotic chromatin condensation and DNA fragmentation by TUNEL and DAPI staining. Scale bar: 50 μm. (B) Identification of apoptotic stages of HA22T cells after ECDT treatment (0, 5, 10, 15 and 20 μM) by annexin V-fluorescein isothiocyanate (FITC)/propidium iodide (PI) analysis. The intensity of early apoptosis showed an increasing trend with an increasing concentration of ECDT. Mock: cells treated with vehicle control (DMSO).

ECDT-induced cytotoxic effects on HA22T cells were confirmed by TUNEL and DAPI staining. Cells undergoing apoptosis were observed from the merged results of TUNEL and DAPI staining, which demonstrated peak accumulations in fluorescence in HA22T cells treated with 20 μM ECDT (Figure 3(A)). To investigate whether ECDT induced apoptosis in HA22T cells, and the numbers of cells in the various stages of apoptosis, ECDT-treated HA22T cells were examined using a flow cytometry-based annexin V-FITC/PI double-staining system. After treatment of HA22T cells with 0, 5, 10, 15 and 20 μM ECDT, the resulting flow cytometry data showed the incidences of early apoptosis/late apoptosis to be 1.05/0.8%, 4.5/2.1%, 11.1/2.8%, 13.4/3.8% and 14.9/5.7%, respectively (Figure 3(B)). The findings revealed the effectiveness and ability of ECDT to trigger early and late apoptosis in HA22T cells.

### ECDT treatment repressed the mitochondrial membrane potential and activated the mitochondrial-dependent apoptotic pathway in HA22T cells

**Figure 4.**
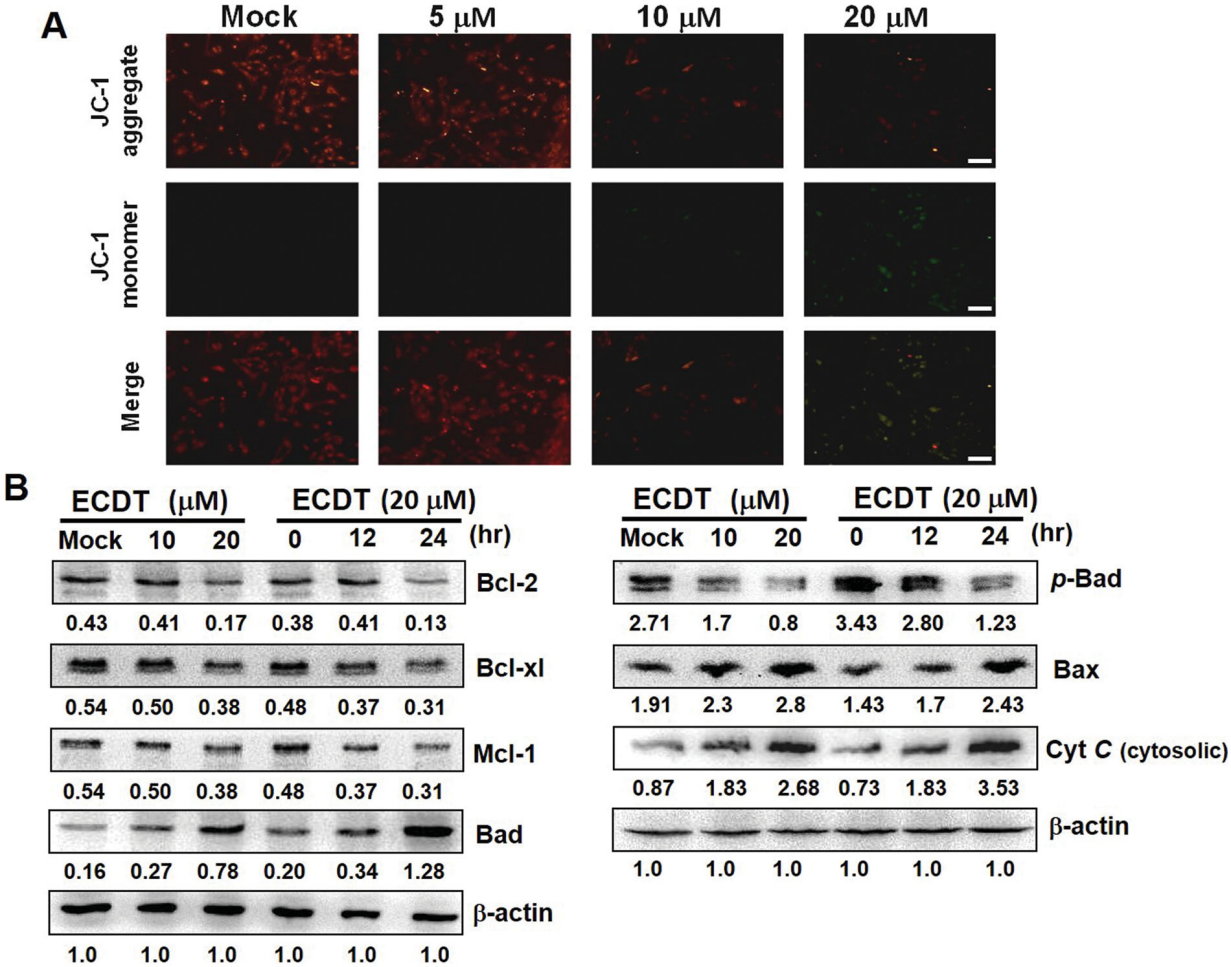
Mitochondrial membrane depolarization and alterations of expression levels of Bcl-2 family caused by ECDT treatment in HA22T cells. (A) ECDT-treated cells stained with JC-1 dye and viewed as red or green under a fluorescence microscope at emission wavelengths of 580 nm and 530 nm. Scale bar: 50 μm. (B) Variations in the expression levels of pro-apoptotic Bax and Bad, and anti-apoptotic Bcl-2, Bcl-xl and Mcl-1, indicated by Western blot. The subsequent release of cytochrome *C* and cytosolic accumulation was expected to trigger ensuing apoptotic pathways, leading to cell death. The cytosol marker, β-actin, was used as the internal control. Mock: cells treated with vehicle control (DMSO).

Loss of the mitochondrial membrane potential (Δ*Ψ*m) is attributed to the mitochondrial disruption that occurs when cells are undergoing the early stages of programmed cell death; this phenomenon is receiving growing attention. JC-1 dye can be employed to detect changes in the mitochondrial membrane potential (Δ*Ψ*m) induced by ECDT treatment. In ECDT-treated HA22T cells, deterioration of mitochondrial health was detected; increased green fluorescence signals and decreased red fluorescence signals observed by fluorescence microscopy showed the significant loss of Δ*Ψ*m caused by ECDT treatment (Figure 4(A)). The intrinsic apoptotic pathway involves activity of mitochondrial members of the Bcl-2 family, which regulates apoptosis by controlling mitochondrial permeability. The results shown in Figure 4(B) demonstrate alteration of the balance in the Bcl-2 family, leading to mitochondrial membrane breakdown after ECDT treatment; the expression levels of Bax, Bad and cytosolic cytochrome C were up-regulated, whereas those of Bcl-2, Bcl-xl and *p*-Bad were down-regulated.

### ECDT induced cell apoptosis through the caspase-dependent pathway

**Figure 5.**
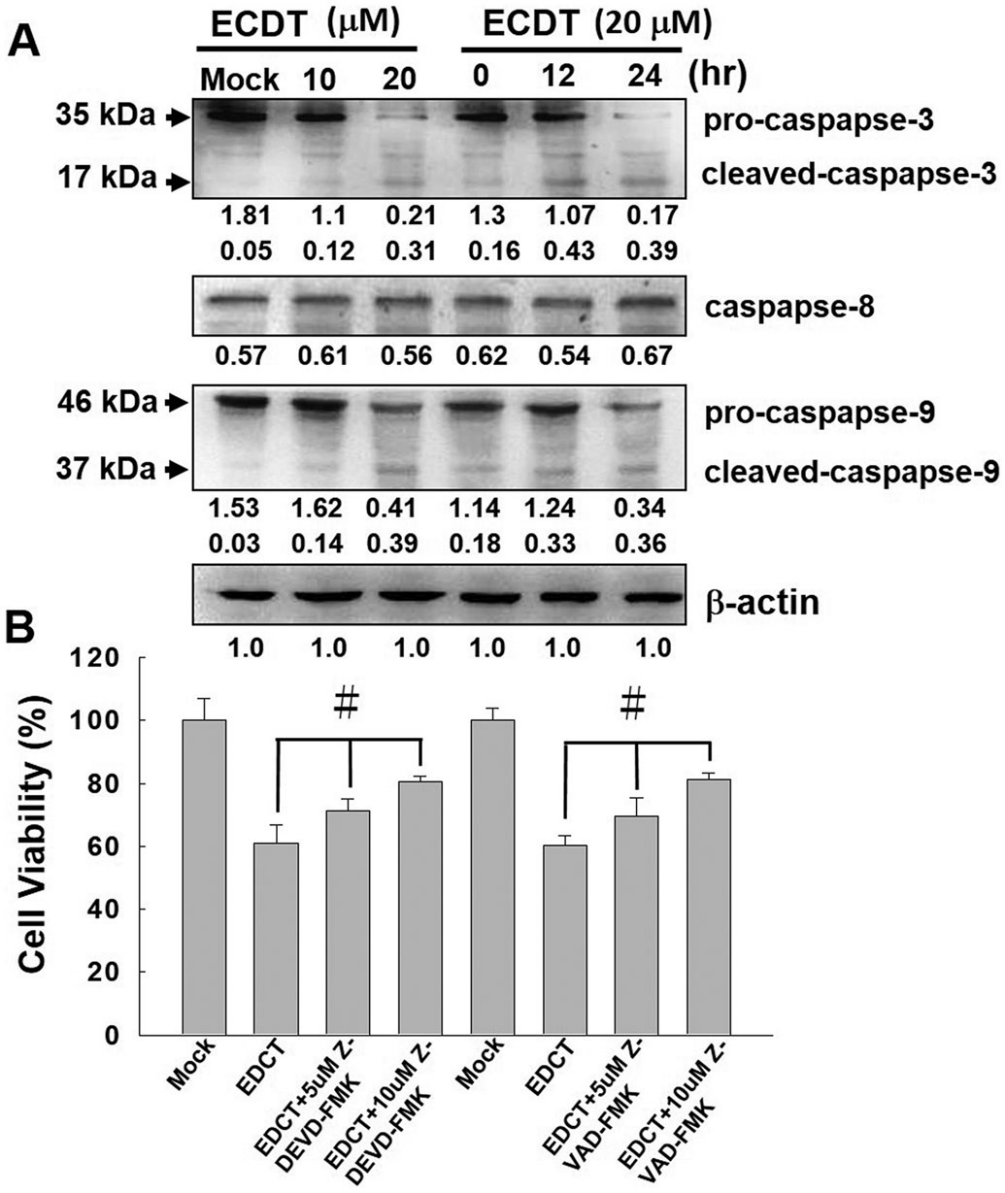
Caspase activation induced by ECDT in HA22T cells. (A) Detection of expression levels of caspases (caspase-3, -8 and -9) in ECDT-treated cells established via Western blot using specific antibodies. Mock: cells treated with vehicle control (DMSO). (B) Prior to ECDT treatment, pan-caspase inhibitors Z-VAD-FMK and Z-DEVD-FMK were applied to cells. Inhibitor assessment was performed in conjunction with evaluation of cell viability in order to verify the data collected via Western blot. The pre-treated cells were harvested at 24 h and subjected to MTT assay. The data show the rescued cell viabilities of the ECDT-treated cells pre-treated with caspase-9 and -3 inhibitors; data were obtained from three independent experiments (^#^*p*< 0.05, compared with the control). Mock: cells treated with vehicle control (DMSO).

Changes in the expression levels of caspases involved in caspase-dependent apoptotic pathways were investigated by Western blot analysis. The levels of caspase-3, caspase-8 and caspase-9 in ECDT-treated cells were examined. As shown in Figure 5(A), the Western blotting data indicated dose- and time-dependent pro-caspase-9 and pro-caspase-3 down-regulation and increases in the expression levels of cleaved-caspase-3 and cleaved-caspase-9; however, the related expression level of caspase-8 remained unchanged. In addition, assessment of cell viability following ECDT treatment of HA22T cells was performed to verify the efficacy of ECDT acting via the caspase-dependent apoptosis pathway. Two caspase inhibitors, Z-VAD-FMK and Z-DEVD-FMK, were employed for cell pre-treatment owing to their cell-permeant property and potent inhibition ability. The data presented in Figure 5(B) validated the results of Western blot analysis, showing that caspase-3 and -9 were engaged during cell apoptosis induced by ECDT.

### ECDT induced the activation of JNK and p38MAPK in HA22T cells

**Figure 6.**
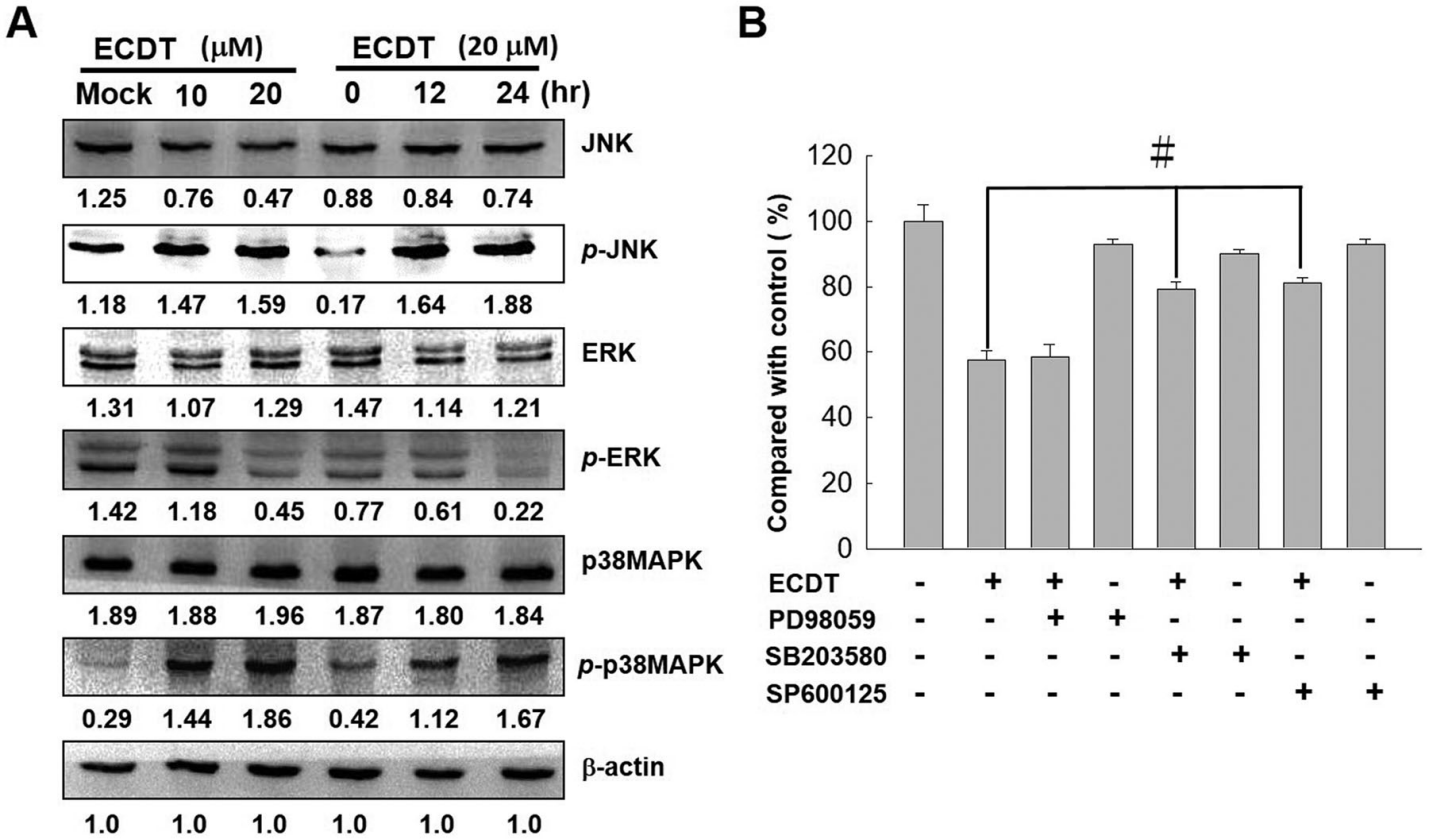
Effects of ECDT on the MAPK pathway. ECDT treatment affected the expression of MAPKs, as shown by Western blot analysis of HA22T cells. Western immunoblots were probed with antibodies against total ERK, *p*-ERK, JNK, *p*-JNK, p38MAPK and *p*-p38MAPK for normalization. (B) MTT assay verifying activation of the MAPK signalling pathway by application of inhibitors of ERK, JNK and p38MAPK individually in ECDT-treated HA22T cells. Mock: cells treated with vehicle control (DMSO).

The activation of three subfamilies (ERK, JNK and p38MAPK) of mitogen-activated protein kinases (MAPKs) by ECDT treatment was examined. The results presented in Figure 6(A) show that the expression levels of *p*-JNK and *p*-p38MAPK were significantly increased, and the phosphorylation activities were up-regulated with an increasing ECDT concentration. These results showed that JNK and p38MAPK were involved in ECDT-induced apoptosis in HA22T cells; therefore, the JNK and p38MAPK signalling pathways are involved in ECDT-induced apoptosis in HA22T cells. Specific inhibitors, SP600125, SB203580 and PD98059, of JNK, p38MAPK and ERK, respectively, were individually employed in MTT assays to verify the activation of the MAPK signalling pathway. As the results presented in Figure 6(B) show, the cell viability was rescued when ECDT-treated cells were pre-treated with inhibitors of JNK and p38MAPK individually, whereas the cell viability remained suppressed when ECDT-treated cells were pre-treated with an inhibitor of ERK. Activation of the JNK and p38MAPK signalling pathways plays an apoptotic role in HA22T cells treated with ECDT.

### Cell viability of ECDT-treated HA22T cells rescued by MAPKs inhibitors

**Figure 7.**
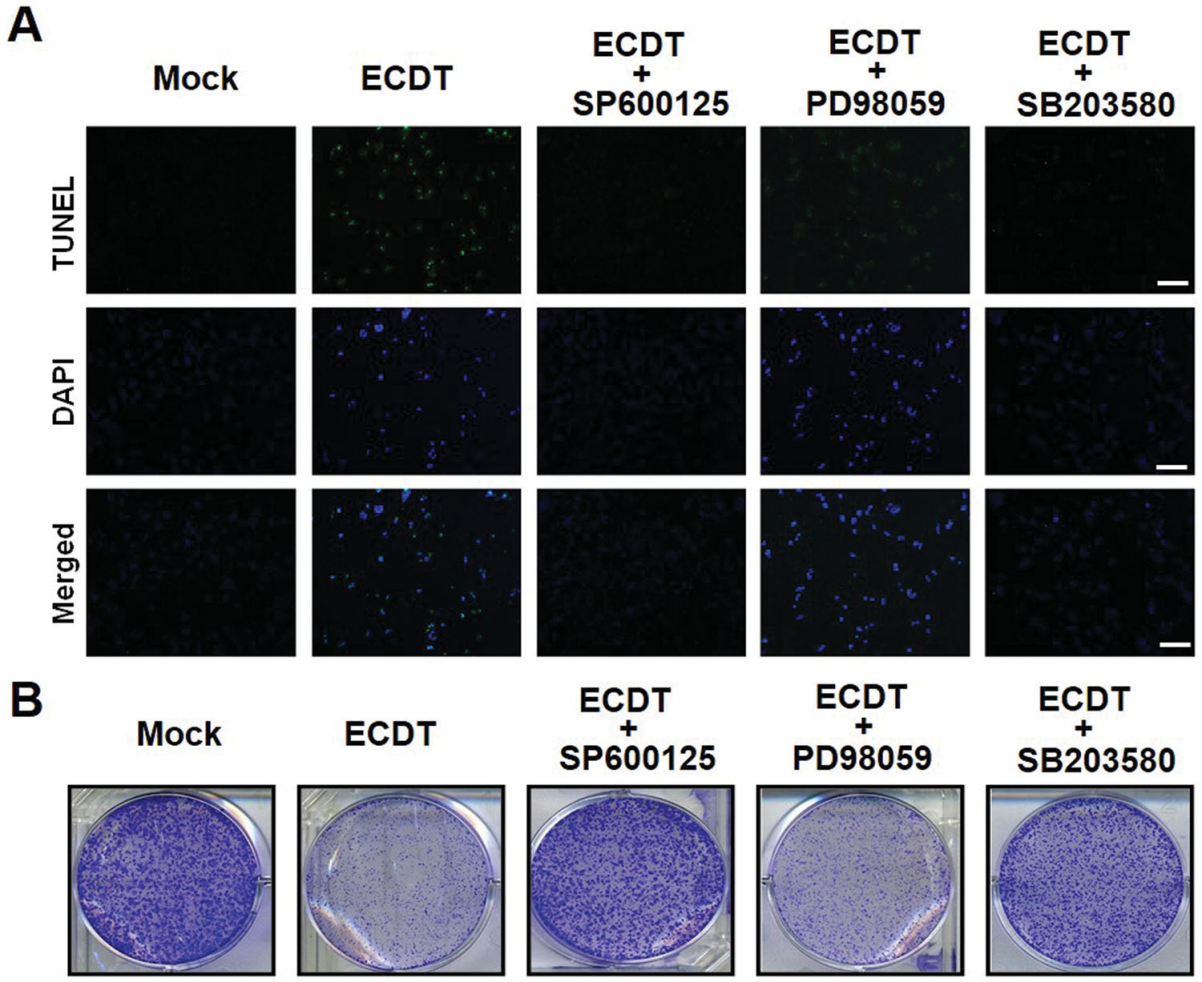
Cell viability of ECDT-treated cells affected by MAPK inhibitors. Three different inhibitors (PD98059: an ERK-specific inhibitor, SB203580: a p38MAPK-specific inhibitor and SP600125: a JNK-specific inhibitor) were used to elucidate MAPKs-activated cell apoptosis upon ECDT treatment in HA22T cells. (A) Fluorescent imaging from TUNEL and DAPI staining indicated ECDT-induced apoptotic characteristics, showing a decrease in the quantity of fluorescence in cells pre-treated with SB203580 and SP600125. (B) A colony formation assay performed to detect cell growth activity of HA22T cells pre-treated with three inhibitors independently showed a rescued cell growth activity of ECDT-treated HA22T cells. The results obtained were representative of three independent experiments (*p*< 0.05 as compared with the control). Mock: cells treated with vehicle control (DMSO).

MAPKs inhibitors (PD98059, SB203580 and SP600125) were individually applied to ECDT-treated cells in TUNEL, DAPI and colony formation assay experiments in order to elucidate the role of the MAPK pathway; the results were analysed by Western blot, as shown in Figure 7(A). The results showed a significant diminishment upon pre-treatment with SB203580 or SP600125 in comparison with the negative control. The data assisted in confirming the partial involvement of the p38MAPK and JNK pathways in ECDT-induced apoptosis of HA22T cells. A similar phenomenon occurred in the colony formation assay, as shown in Figure 7(B); the numbers of ECDT-treated cells pre-treated with inhibitors of JNK and p38MAPK independently were higher than the number of ECDT-treated cells with no addition of inhibitors.

## Discussion

Plant-derived herbal medicines found to possess biochemical activities have long been adopted for clinical and pharmaceutical purposes. *M. charantia*, popularly known as bitter melon, is an important market vegetable consumed prevalently in Asia (Nerurkar and Ray 2010; Li et al. 2012). One powerful activity of this plant, apart from it containing essential nutrients required by humans, is to reduce the blood sugar level (Joseph and Jini 2013). Therefore, multiple investigations of proteins or metabolites in *M. charantia* were conducted, which demonstrated its hypoglycaemic effect, in addition to antitumor and antiviral activities, which have been well-documented worldwide (Fang and Ng 2011; Wang and Ma 2014). For example, momorcharins and Kuguacin J in *M. charantia* each possess a notable ability to inhibit human prostate cancer cells (Xiong et al. 2009; Pitchakarn et al. 2011). 5β,19-Epoxycucurbita-6,23(*E*)-diene-3β,19(*R*),25-triol (ECDT) isolated from *M. charantia* was found to exert cytotoxicity towards liver cancer cells (HA22T), and this anticancer activity was attributed to its action on the induction of programmed cell death.

### ECDT induces apoptosis in HA22T cells, leading to cell death

Treatment of HCC cell line HA22T with ECDT isolated from *M. charantia* was found to exert cell cytotoxic effects. The intensity of the ECDT dose had a positive correlation with the cytotoxic effect on HA22T cells. A wound healing assay was performed to assess directional cell migration. The migration rate of cells was reduced with treatment with increased concentrations of ECDT, implying that cells lost their migration activity under a higher concentration of ECDT. The effectiveness of the cytotoxic influence of ECDT on HA22T cells was elucidated from data collected via the assays mentioned above. Further precise bioassays would demonstrate the intracellular changes of cells responding biochemically and physiologically to the addition of ECDT. The apoptotic morphological characteristics of ECDT-treated cells after TUNEL/DAPI staining demonstrated an increase in fluorescence-labelled DNA blunt ends, in addition to cell shrinkage and blebbing, with respect to the control. Both assays showed extensive DNA degradation when cells were treated with ECDT, which subsequently led to cell death. Furthermore, during the onset of apoptosis, phosphatidylserines (PS) that are normally actively held facing the inner side of the cell membrane were translocated to the external side of the cell membrane. Exposure of PS on the outer surface of the cell forms the basis of annexin V binding, allowing identification of the earlier stage of cell apoptosis. When the annexin V assay was coupled with PI staining, the cellular DNA of apoptotic cells in the early and late stages of the process treated with ECDT was labelled, as shown in Figure 3(A).

### ECDT induced mitochondrial dysfunction in HA22T cells, leading to apoptosis

In regard to the possibility of a characteristic related to the induction of cell apoptosis, mitochondrial dysfunction, following ECDT treatment of HA22T cells, the mitochondrial membrane potential (Δ*Ψ*m) was assessed using JC-1 dye. By means of JC-1 application, apoptotic cells were easily distinguished from non-apoptotic cells. As shown in Figure 4(A), discrimination of apoptotic and non-apoptotic mitochondria in ECDT-treated cells was facilitated by JC-1 dye, resulting in the appearance of increased green fluorescence signals and decreased red signals, suggesting mitochondrial membrane depolarization. Therefore, to explore whether the launch of the mitochondria-dependent apoptotic pathway was induced by ECDT, investigation of the differentiated expression levels of mitochondria-related apoptosis proteins was performed via Western blot. As shown in Figure 4(B), the differentiated expression of Bcl-2 family members and an increase in cytochrome *C* release apparently signalled commencement of the ensuing caspase-dependent apoptotic pathway (Ola et al. 2011). The release of cytochrome *C* from mitochondria, regulated by the relative amounts of proapoptotic (Bax, Bak) and antiapoptotic Bcl-2 proteins (Bcl-2, Bcl-xl), activates a caspase cascade reaction that degrades other cellular targets, leading to cell death (Martinou and Youle 2011). The Bax/Bcl-2 ratio determines the susceptibility to apoptosis and adjusts the release of cytochrome *C* from mitochondria into the cytosol (Yang et al. 2006; Kilbride and Prehn 2013).

Caspases have been identified as indispensable mediators of the induction of proteolytic activation for apoptotic chromatin condensation and DNA fragmentation. Activation of caspase-9 was reported to be a signal of mitochondria-mediated apoptosis that initiates activation of caspase-3, whereas caspase-8 is activated in response to extracellular apoptosis-inducing ligands (Ola et al. 2011). Caspase-3 is activated by caspase-8 or caspase-9 through the extrinsic or intrinsic apoptotic pathway, respectively (Affar et al. 2001). Therefore, to discern the received proapoptotic signals, the expression levels of caspase-3, caspase-9 and caspase-8 upon ECDT treatment in HA22T cells were studied. By Western blot analysis, the expression levels of cleaved-caspase-3 and cleaved-caspase-9 were found to be up-regulated in ECDT-treated cells, while the activity of caspase-8 remained the same (Figure 5(A)). The data revealed that the HA22T cell apoptotic pathway induced by ECDT is stimulated through differentiated caspase activity involving mitochondrial stress, other than the activation associated with the cytoplasmic death domain initiated by cell surface ligands. The apoptotic signals result in a breakdown of PARP-1 by caspases and collapse of DNA in the nucleus, which eventually leads to cell death (Wang et al. 2006). Additionally, caspase inhibitor assessment using Z-VAD-FMK and Z-DEVD-FMK was performed concurrently with ECDT treatment in HA22T cells. The results showed that caspase-3 and -9 inhibitors rescued the cell viability from ECDT-induced cell cytotoxicity (Figure 5(B)). With the aid of inhibitor assessment, the information collected confirmed that apoptosis induced by ECDT in HA22T cells is mediated through the mitochondrial-dependent and caspase-dependent apoptotic pathways.

### Involvement of MAPK apoptotic signalling pathway induced by ECDT

The MAPK signalling pathway comprises activation of the p38MAPK and JNK pathways in response to various chemicals and stresses during apoptosis (Coulthard et al. 2009). The dynamic balance between ERK and the JNK and p38MAPK pathways is critical in determining whether the cell will die or survive. As JNKs and p38MAPKs are important stress-responsive proteins, whereas ERKs are crucial for cell survival, activation of the p38MAPK and JNK pathways and concurrent inhibition of ERK contribute to cell apoptosis, as shown in several studies (Yanase et al. 2002). Moreover, cell apoptosis activated through the involvement of p53 (tumour suppressor protein) modulation has been reported to be controlled by chemical stress-induced p38 and JNK activation (Erdogan and Turkekul 2020). In response to abnormal proliferative signals, the tumour suppressive function of p53 is set to check the passage through the cell cycle and coordinate the machinery of cell apoptosis. Signalling between p53 and Bcl-2 has been evidenced as a strong genetic and biochemical tie that is of fundamental importance in cancer biology. p53 protein in its part acts to decrease the antiapoptotic Bcl-2 expression level and increase the proapoptotic Bax protein expression (Cui et al. 2012). Further biochemical assays (such as TUNEL, DAPI and cell colony formation assays, Figure 6) were performed with the addition of inhibitors of JNK, P38 and ERK individually to verify the credibility of the analytical results from Western blot. All results indicated that the MAPK signalling pathway played a part in ECDT-induced apoptosis of HA22T cells.

## Conclusions

The promising findings of this study showed that a sequence of the death message was transduced via p38MAP/JNK-, mitochondria- and caspase-dependent pathways in ECDT-induced apoptosis of HA22T cancer cells. Among a variety of processes involved in the complex physiological and biochemical mechanistic connections within the cell, cell death deployment other than apoptosis is probably engaged in response to stress stimuli of HA22T cancer cells by ECDT. On the whole, each component of the biochemical experimental design implemented in this study served to provide supporting evidence to corroborate that ECDT isolated from *M. charantia* is capable of initiating anticancer actions through mitochondria- and caspase-dependent apoptotic pathways, and via p38MAPK and JNK activation. Therefore, investigation of the anticancer property of ECDT may shed light on a variety of future directions with regards to its pharmaceutical and clinical prospects as a potential chemotherapeutic agent.

## Author contributions

Y.-J. W. and C.-I. C. conceived and designed the experiments. C.-I. C. isolated and identified the compound. M.-K. Y., C.-R. C. and W.-T. W. performed the experiments and analysed the data. M.-K. Y. and Y.-J. W. wrote the paper. All authors read and approved the final manuscript.
